# An Unrecognized Fundamental Relationship between Neurotransmitters: Glutamate Protects against Catecholamine Oxidation

**DOI:** 10.3390/antiox10101564

**Published:** 2021-09-30

**Authors:** Wenping Wang, Ximing Wu, Chung S. Yang, Jinsong Zhang

**Affiliations:** 1 State Key Laboratory of Tea Plant Biology and Utilization, School of Tea & Food Science, Anhui Agricultural University, Hefei 230036, China; wpw@ahau.edu.cn (W.W.); WXM@ahau.edu.cn (X.W.); csyang@pharmacy.rutgers.edu (C.S.Y.); 2 Department of Chemical Biology, Ernest Mario School of Pharmacy, Rutgers, The State University of New Jersey, Piscataway, NJ 08854, USA; 3 International Joint Research Laboratory of Tea Chemistry and Health Effects, Anhui Agricultural University, Hefei 230036, China

**Keywords:** catecholamines, dopamine, glutamate, neurotransmitters, oxidation

## Abstract

Neurotransmitter catecholamines (dopamine, epinephrine, and norepinephrine) are liable to undergo oxidation, which copper is deeply involved in. Catecholamine oxidation-derived neurotoxicity is recognized as a pivotal pathological mechanism in neurodegenerative diseases. Glutamate, as an excitatory neurotransmitter, is enriched in the brain at extremely high concentrations. However, the chemical biology relationship of these two classes of neurotransmitters remains largely unknown. In the present study, we assessed the influences of glutamate on the autoxidation of catecholamines, the copper- and copper-containing ceruloplasmin-mediated oxidation of catecholamines, the catecholamine-induced formation of quinoprotein, catecholamine/copper-induced hydroxyl radicals, and DNA damage in vitro. The results demonstrate that glutamate, at a physiologically achievable molar ratio of glutamate/catecholamines, has a pronounced inhibitory effect on catecholamine oxidation, catecholamine oxidation-evoked hydroxyl radicals, quinoprotein, and DNA damage. The protective mechanism of glutamate against catecholamine oxidation could be attributed to its restriction of the redox activity of copper via chelation. This previously unrecognized link between glutamate, catecholamines, and copper suggests that neurodegenerative disorders may occur and develop once the built-in equilibrium is disrupted and brings new insight into developing more effective prevention and treatment strategies for neurodegenerative diseases.

## 1. Introduction

Catecholamines, such as dopamine and norepinephrine, take part in regulating a variety of mental processes, including cognitive ability, attention, memory, mood, and reward [1,2,3,4,5]. Catecholamines have adjacent hydroxyl groups on their benzene rings [6], thus making them susceptible to autoxidation, and produce hydrogen peroxide [7,8,9], semiquinone anion radicals [8,10], and quinones [11,12]. Quinones further initiate intramolecular cyclization to form the end products of neuromelanin polymers [13,14,15,16,17]. All these intermediates and end products may be toxic to neuron cells, and thus the autoxidation of catecholamines is considered to be an important mechanism of neuron cell loss in Parkinson’s disease [18,19,20,21]. Moreover, the disorder of copper homeostasis is involved in neurological diseases, such as Parkinson’s disease [22,23]. Redox-active copper can facilitate the oxidation of catecholamines via the formation of hydroxyl radicals [24,25]. It is known that DNA is an especially sensitive site within the cell for copper-mediated damage because copper ions have a high affinity for DNA, forming a DNA–Cu complex [26,27]. Catecholamines and DNA-localized copper can cause DNA damage via the site-specific attack of hydroxyl radicals on DNA [25,26].

Glutamate, as a neurotransmitter, plays an important role in learning, memory, neuronal plasticity, and brain development [28,29,30,31,32]. The excessive stimulation of glutamate receptors causes the excitatory toxicity of neuron cells [33,34]; thus, neurons are endowed with high-affinity glutamate transporters to enrich glutamate [35]. Consequently, extracellular glutamate concentrations, or interstitial fluid glutamate concentrations, are maintained in levels as low as 0.5–5 μM [36], and intracellular concentrations of glutamate reach as high as 6–12 mM [35]. In contrast, the intracellular concentrations of dopamine, the major catecholamine neurotransmitter in dopaminergic neurons, are only at the level of 0.05–0.1 mM [37,38,39]. The present study investigated whether glutamate has an impact on catecholamine oxidation in vitro. We found that glutamate was able to prevent the autoxidation of catecholamines and autoxidation-associated quinoprotein formation, the copper-mediated oxidation of catecholamines, catecholamine/copper-triggered DNA damage, and quinoprotein formation.

## 2. Materials and Methods

### 2.1. Chemicals and Drugs

Dopamine (hydrochloride) (CAS. No: 28094-15-7) was purchased from Solarbio Science & Technology Co., Ltd. (Beijing, China). Norepinephrine (CAS. No: 51-41-2), epinephrine (CAS. No: 51-43-4), and glutamate (CAS. No: 6893-26-1) were obtained from Macklin Biochemical Co., Ltd. (Shanghai, China). 3, 4-dihydroxyphenylacetaldehyde (DOPAL) (CAS. No: 5707-55-1) was a product of Santa Cruz Biotech (Dallas, TX, USA). Ceruloplasmin (CAS. No: 9031-37-2), monoamine oxidase (MAO) (M7316), 2’,7’-dichlorofluorescin diacetate (DCFH-DA) (CAS. No: 4091-99-0), 3-hydroxycinnamic acid (3-CCA) (CAS. No: 531-81-7), dithiothreitol (CAS. No: 3483-12-3), glyceraldehyde-3-phosphate dehydrogenase (GAPDH) (CAS. No: 9001-50-7), and nitroblue tetrazolium (NBT) (CAS. No: 298-83-9) were purchased from Sigma (St. Louis, MO, USA). The polyvinylidene difluoride (PVDF) membrane was a product of Bio-Rad Laboratories, Inc. (Hercules, CA, USA). Plasmid pBR322 DNA (#N3033L) was obtained from New England Biolabs (Beijing, China). Other chemicals used were of the highest grade available.

### 2.2. Dopamine Oxidation Assessment

Dopamine was incubated in a 0.15 M, pH 7.4 phosphate buffer solution (PBS) at 37 °C. With the increase in oxidation time, the dopamine solution gradually oxidized to yellow. According to the results of spectral scanning, an absorption value of 410 nm was selected to characterize the oxidation degree of the dopamine. Kinetic alterations were recorded using a plate reader (Molecular Devices SpectraMax M2e, Sunnyvale, CA, USA).

### 2.3. Detection of Reactive Oxygen Species

To detect reactive oxygen species, dopamine was incubated in PBS (0.15 M, pH 7.4) containing 50 μM of DCFH-DA at 37 °C. Dynamic changes in fluorescence intensity were recorded using the aforementioned plate reader at an excitation wavelength of 488 nm and an emission wavelength of 525 nm. 

### 2.4. Detection of Hydroxyl Radicals

The chemicals were co-incubated with 3 mM of 3-CCA as a fluorescent probe at 37 °C. The dynamic curve of the fluorescence intensity was recorded using the aforementioned plate reader. The excitation and emission wavelengths were 388 nm and 446 nm, respectively. 

### 2.5. Detection of DNA Damage 

DNA cleavage was evaluated by agarose gel electrophoresis using pBR322 plasmid DNA. The DNA and chemicals were mixed in 0.15 M PBS (pH 7.4) at a final volume of 50 μL and incubated at 37 °C. The samples were loaded by 6 × DNA loading buffer in 1% agarose gel containing 40 mM of Tris-acetate and 1 mM of EDTA (pH 8.0) as well as Super/Gel Red (Tiangen Biotech, Beijing, China). In 1 × TAE gel buffer, electrophoresis was carried out on a horizontal plate gel apparatus under 90 V conditions for 1 h. 

### 2.6. NBT/Glycinate Redox-Cycling Staining of Quinoprotein 

The chemicals and GAPDH were mixed in 0.15 M PBS (pH 7.4) at 37 °C and the reaction was terminated by adding dithiothreitol. Then, 10% SDS polyacrylamide gels were used for second-dimension electrophoresis. The proteins on the gel were transferred to a methanol-soaked PVDF membrane in an electrophoresis transfer solution. The quinoproteins were stained by redox-cycling staining [40,41]. Briefly, the membrane was incubated with 0.24 mM NBT in 2 M potassium glycinate (pH 10.0) in the dark to form purple bands, then the stained membrane was rinsed with distilled water. 

### 2.7. Dopamine Retention Detected by High Performance Liquid Chromatography (HPLC)

Dopamine was quantitatively detected by HPLC (Waters Instruments, Inc., Rochester, MN). Chromatographic separation was performed on a Gemini 5 u C18 110 A column (250 × 4.60 mm, Phenomenex Inc., Torrance, CA, USA). The separation conditions were as follows: the column temperature was maintained at 30 °C, the flow rate was 1 mL/min, the single injection volume was 10 μL, and the mobile phases were (A) 0.15% acetic acid–water (ultra-pure water plus 0.15% acetic acid) and (B) acetonitrile. A gradient elution separation method was set, the total detection time was 40 min, and phase A was taken as an example: (1) Initial mobile phase proportion A: 85%, keep running for 10 min; (2) switch to another gradient elution A: 77%, run for 15 min; (3) reduce to A: 71% within 1 min; (4) convert A: 0% at the 26th min and maintain for 4 min; (5) return to initial state A: 85% within 2 min and maintain for 8 min; (6) switch to A: 80% at 40 min and maintain the balance system for the next injection. The amount of dopamine in the eluent was measured by the absorbance with wavelengths of 278 nm. 

### 2.8. Determination of Ceruloplasmin Ferrous Oxidase Activity

Ceruloplasmin ferrous oxidase activity was determined using a kit purchased from Jiancheng Bioengineering Institute (Nanjing, China). 

## 3. Results and Discussion

### 3.1. Autoxidation of Dopamine and the Protective Role of Glutamate

**Figure 1:**
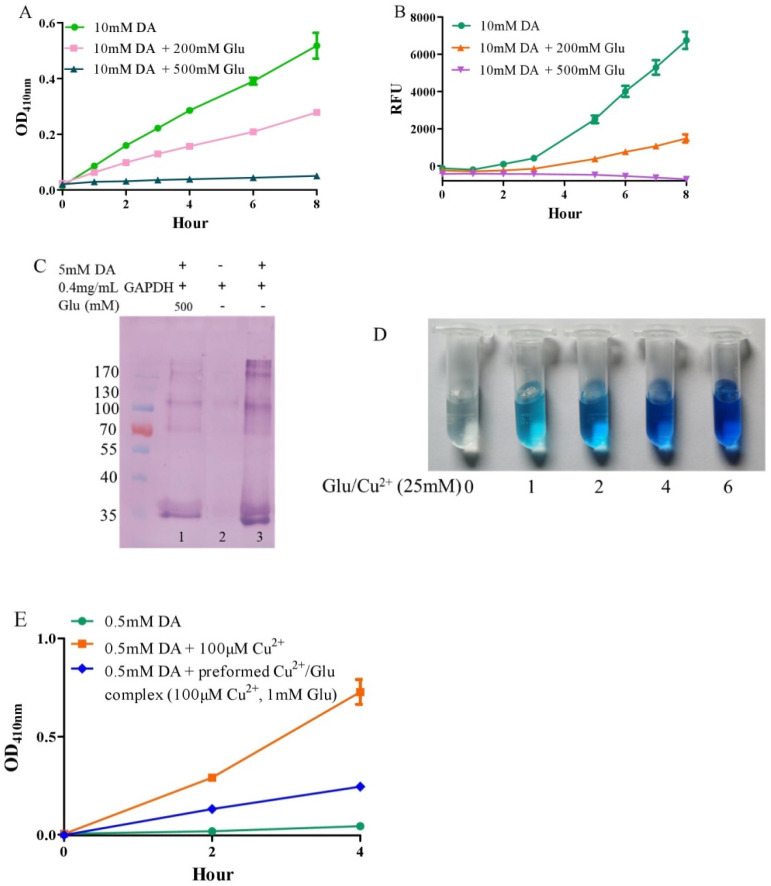
Autoxidation of dopamine and the protective role of glutamate. (**A**) Dopamine autoxidationmeasured by OD_410 nm_. (**B**) Dopamine-caused ROS production detected by DCFH-DA. (**C**) Dopamine-caused quinoprotein in GAPDH. (**D**) Glutamate-copper complexes. (**E**) Influence of free copper and preformed copper-glutamate complex on dopamine oxidation. Chemicals were mixed in 0.15 M PBS (pH 7.4) and incubated at 37 °C for indicated time or 1 h for C. Data are presented as mean ± range (*n* = 2). Note: DA: Dopamine, Glu: Glutamate.

Under alkaline and aerobic conditions, dopamine can undergo autoxidation to form quinones and produce hydrogen peroxide [9,42]. The quinones further polymerize to form macromolecular neuromelanin polymers, which increase the pigmentation of neurons in the substantia nigra [43]. The pigmented dopaminergic neurons in the substantia nigra are preferentially lost in Parkinson’s disease [13]. In the present study, 10 mM of dopamine was used to observe time-dependent color development due to dopamine oxidation under physiological conditions (a pH 7.4, 0.15 M PBS). Compared to dopamine alone, the addition of −200 mM of glutamate effectively inhibited dopamine oxidation, while that of −500 mM of glutamate almost completely inhibited the oxidation, as evaluated by the color development at OD410nm (Figure 1A). Since dopamine autoxidation leads to the formation of reactive oxygen species (ROS), 50 μM of DCFH-DA as a fluorescent probe was used to detect the ROS produced by 10 mM of dopamine. The production of ROS was significantly inhibited by −200 mM of glutamate, while 500 mM of glutamate nearly entirely inhibited ROS production (Figure 1B). During the nonenzymatic autoxidation process of dopamine, intermediate quinones can covalently react with cysteine sulfhydryl groups in proteins or enzymes, leading to the formation of quinoproteins [8,44,45]. Quinoprotein adduct formation may play a role in the age-dependent selective vulnerability of dopaminergic neurons in Parkinson’s disease [46]. To detect the production of dopamine-quinoproteins, dopamine and GAPDH (with a free thiol group of a cysteine residue at position 151) were co-incubated at 37 °C for 1 h in vitro. As shown in Figure 1C, in the absence of dopamine, quinoproteins were not observed (lane 2). After a 1 h co-incubation of GAPDH and dopamine (5 mM), dopamine-quinoproteins could be clearly visualized (lane 3). The addition of glutamate (500 mM, lane 1) sufficiently inhibited quinoprotein formation. These results together suggest that glutamate has an inhibitory effect on the autoxidation of dopamine. However, the specific mechanism involved is not clear. We speculated that this may involve the nature of autoxidation. In fact, the spin limitation of dioxygen is a dynamic barrier to the oxidation of biomolecules such as dopamine [47]. The direct reaction between the two requires a large amount of activation energy; thus, the oxidation rate of biomolecules is very slow and the real autoxidation of biomolecules is a negligible process [47]. However, many transition metals with various spin states can overcome the spin limitation of dioxygen and thus increase the oxidation rate of biomolecules. The commonly described autoxidation of biomolecules such as catecholamines is actually promoted by transition metals [47]. David et al. proposed that the oxidation of dopamine in the absence of added copper may be significantly influenced by the presence of metal impurities [48]. In deionized water that was further purified by chromatography over Chelex 100 resin prior to use, epinephrine does not autoxidize. However, epinephrine was oxidized rapidly in deionized water, but this oxidation could be prevented by desferal (a potent metal chelating agent) [49]. The autoxidation of (-)-epigallocatechin-3-gallate, a well-documented redox-active catechin mainly found in green tea, can be largely prevented by EDTA, indicating that trace amounts of transition metals are involved in the autoxidation process [50]. Many buffer systems, especially phosphate, can form complexes with transition metals. Thus, in many experimental systems, the presence of trace metals in the buffer is inevitable [47]. Put simply, there is no pure “autoxidation”. The essence of biomolecule autoxidation is oxidation involving trace amounts of transition metals. It has been reported that glutamate can react with copper by forming complexes [51,52]. Indeed, as shown in Figure 1D, the characteristic blue color of glutamate-copper complexes is enhanced as a function of the increased glutamate concentrations. Preformed glutamate-copper complexes consistently show a compromised capacity to promote dopamine oxidation as compared with free copper (Figure 1E). We thus speculate that the inhibitory effect of glutamate on the autoxidation of dopamine could be attributed to glutamate’s restriction on redox-active copper bound to dopamine or the buffer system. Since trace amounts of transition metals, which promote the autoxidation of biomolecules, form complexes with biomolecules and/or buffers, as much as 14 mM of EDTA was required to effectively suppress the “autoxidation” of (-)-epigallocatechin-3-gallate [50]. The present study also consistently showed that higher molar ratios of glutamate/dopamine were needed to fully inhibit the autoxidation of dopamine. In the case of the free copper-promoted oxidation of dopamine, which has been implicated in dopamine-associated toxicity [11,53], we estimated that glutamate would be highly effective in preventing dopamine oxidation—i.e., to achieve an effective protective effect, the molar ratios of glutamate/dopamine are significantly lowered compared to the case of dopamine “autoxidation”. Next, we examined this possibility.

### 3.2. Glutamate Protects against Copper-Facilitated Dopamine Oxidation

**Figure 2:**
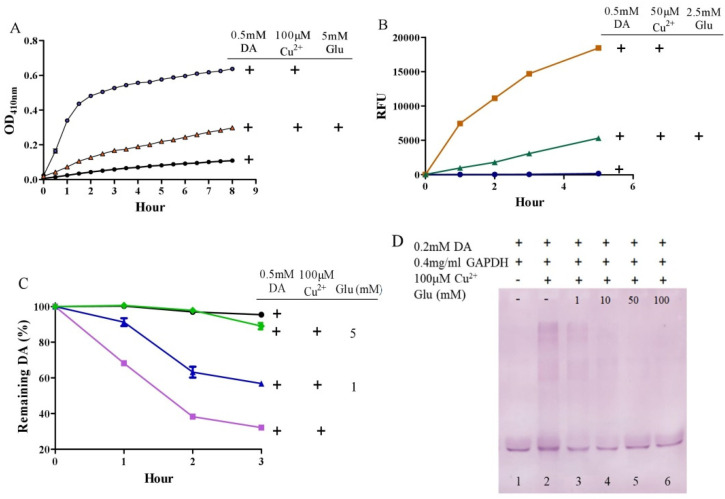
Glutamate protects against copper-induced dopamine oxidation. (**A**) Dopamine oxidation measured by OD_410 nm_. (**B**) Hydroxyl radicals detected by 3-CCA. (**C**) Dopamine levels detected by HPLC. (**D**) Dopamine-caused quinoprotein in GAPDH. Chemicals were mixed in 0.15 M PBS (pH 7.4) and incubated at 37 °C for indicated time or 20 min for D. Data are presented as mean ± range (*n* = 2). Note: DA: Dopamine, Glu: Glutamate.

It is surprising that substantial concentrations of both dopamine and copper (0.4 mM) coexist in the substantia nigra, although the precise free copper concentration is not known [48,54]. Moreover, many studies have shown that copper levels are elevated in the cerebrospinal fluid (CSF) of patients with Parkinson’s disease [55,56]. Copper can facilitate dopamine oxidation and meanwhile leads to the production of highly active hydroxyl radicals and, accordingly, DNA damage [25,57]. These processes may contribute to the observed loss of dopaminergic neurons in patients with Parkinson’s disease [58]. Therefore, we further evaluated the influence of glutamate on copper-accelerated dopamine oxidation. As shown in Figure 2A, compared with 0.5 mM of dopamine alone, the addition of 100 μM of copper hugely promoted the oxidation of dopamine. Glutamate at a concentration of 5 mM effectively suppressed copper-promoted dopamine oxidation (Figure 2A). Concerning hydroxyl radical production by copper and dopamine, we used a hydroxyl radical-specific probe, the 3-CCA, for the assessment. In a redox system of 0.5 mM of dopamine and 50 μM of copper, hydroxyl radical production was clearly sensed by the 3-CCA. In this redox system, the addition of glutamate at a concentration of only 2.5 mM substantially inhibited hydroxyl radical production (Figure 2B). In addition, we used HPLC to detect dopamine retention. As shown in Figure 2C, the copper facilitated dopamine oxidation in a time-dependent manner. After 3-h incubation of copper and 0.5 mM of dopamine, about 30% of the dopamine remained. At the same time, it was observed that the glutamate inhibited the copper-mediated dopamine oxidation in a dose-dependent manner. Specifically, the retention of dopamine could be effectively increased by as low as 1 mM of glutamate, and near-complete retention of dopamine could be achieved by 5 mM of glutamate (Figure 2C). We further characterized the influence of glutamate on dopamine-initiated quinoprotein formation (Figure 2D). Following a 20-min incubation of 0.2 mM of dopamine and GADPH, quinoproteins were hardly detected (lane 1). Under the circumstances, copper markedly promoted quinoprotein formation (lane 2). Nonetheless, glutamate was able to dose-dependently inhibit copper-initiated quinoprotein formation. A low concentration of glutamate (1 mM) could be significantly effective (lane 3), while 10 mM of glutamate almost completely inhibited copper-initiated quinoprotein formation (lane 4). Altogether, these four lines of evidence clearly demonstrate that glutamate is highly effective in inhibiting copper-facilitated dopamine oxidation.

### 3.3. Glutamate Protects against Ceruloplasmin-Facilitated Dopamine Oxidation and Dopamine Oxidation-Caused Modification of Ceruloplamin

**Figure 3:**
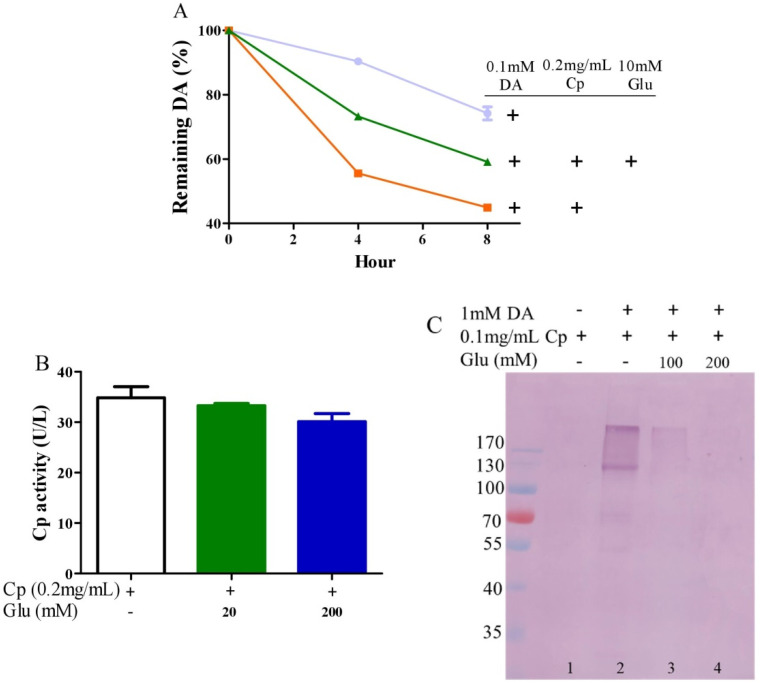
Glutamate protects against ceruloplasmin-promoted dopamine oxidation. (**A**) Dopamine levels detected by HPLC. (**B**) Ferrous oxidase activity of ceruloplasmin. (**C**) Dopamine-caused quinoprotein in ceruloplasmin. All reactions were conducted in 0.15 M PBS (pH 7.4) and incubated at 37 °C for indicated time or 1 h for (**C**). Data are presented as mean ± range (*n* = 2). Note: DA: Dopamine, Glu: Glutamate, Cp: Ceruloplasmin.

Ceruloplasmin, as expressed in human brain glial cells [59,60], is a ferrous oxidase. Ceruloplasmin plays an important role in iron homeostasis by oxidizing toxic ferrous iron so as to favor the strong binding of ferric iron to serum transferrin [61,62,63]. Ceruloplasmin with six copper atoms [64,65] also catalyzes the oxidation of catecholamines [24,49,66,67,68]. Epinephrine oxidation rates enhanced by ceruloplasmin can be slowed down by a metal chelating agent [49], suggesting that copper bound to either epinephrine or ceruloplasmin is probably involved in this catalytic reaction. We thus inferred that glutamate would be able to restrict ceruloplasmin-facilitated dopamine oxidation by forming complexes with copper. To examine this possibility, we measured dopamine oxidation catalyzed by ceruloplasmin and investigated the potential impact of glutamate on dopamine oxidation catalyzed by ceruloplasmin using HPLC. Ceruloplasmin (equivalent to 5 μM of copper) promoted the oxidation of 0.1 mM of dopamine, while 10 mM of glutamate inhibited the ceruloplasmin-catalyzed oxidation of dopamine (Figure 3A). Despite the fact that ceruloplasmin ferrous oxidase can be suppressed by a metal chelating agent such as EDTA [69], glutamate at levels that suppressed the dopamine oxidation activity of ceruloplasmin (Figure 3A) did not affect the activity of ceruloplasmin ferrous oxidase (Figure 3B). This is probably due to the different manner of copper dependence in the two types of activity. Importantly, we found that ceruloplasmin-triggered dopamine oxidation, in turn, caused the quinonization of ceruloplasmin with the formation of quinoproteins. As shown in Figure 3C, in the absence of dopamine, quinoproteins were unable to be detected from the ceruloplasmin (lane 1). In the presence of 1 mM of dopamine, the quinonization of the ceruloplasmin was salient (lane 2). Nonetheless, 100–200 mM of glutamate was highly effective in protecting against the quinonization of the ceruloplasmin (lane 3, 4). Dopamine-caused quinonization of ceruloplasmin suggests that (1) oxidized products of dopamine generated from ceruloplasmin include highly active and thus harmful quinones, and (2) the reciprocal interaction of dopamine and ceruloplasmin may impair the ferrous oxidase activity of ceruloplasmin due to quinonization, thus increasing the accumulation of ferrous ion, leading to hydroxyl radical-associated oxidative stress. Decreased ceruloplasmin levels are associated with an earlier onset of Parkinson’s disease [70,71,72]. Many studies have observed low ceruloplasmin ferrous oxidase activity in the substantia nigra and CSF of Parkinson’s disease patients [73,74,75]. However, the relevant molecular mechanism remains elusive. The interplay of dopamine and ceruloplasmin firstly identified herein may be responsible, at least in part, for the loss of the ferrous oxidase activity of ceruloplasmin. Fortunately, the reciprocal interaction of dopamine and ceruloplasmin, with a loss at both sides, can be effectively halted by glutamate.

### 3.4. Glutamate Inhibits Copper/Catecholamine-Induced DNA Damage

**Figure 4:**
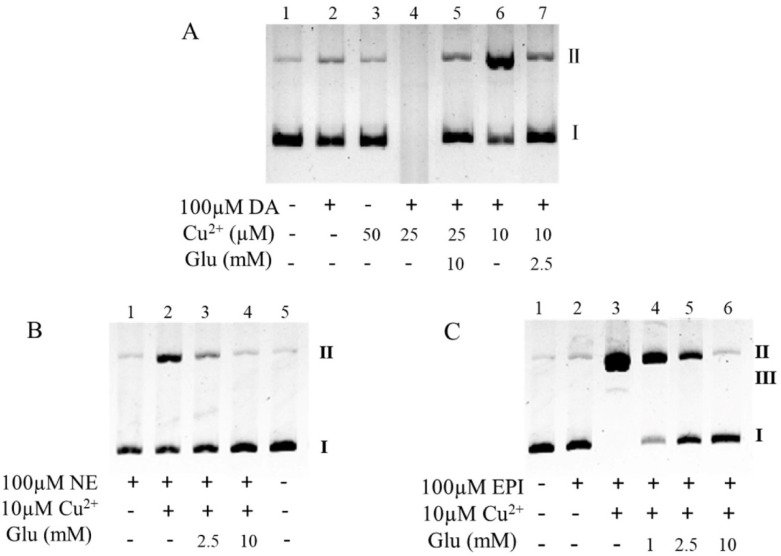
Glutamate protects against copper/catecholamine-induced DNA damage. (**A**) Dopamine. (**B**) Norepinephrine. (**C**) Epinephrine. Chemicals were mixed in 0.15 M PBS (pH 7.4) and incubated at 37 °C for 1 h. All reaction mixtures contained 10 μg/mL plasmid DNA. DNA structures: I, closed circular double-stranded supercoiled DNA; II, relaxed open circle DNA; and III, linear DNA. Note: DA: Dopamine, Glu: Glutamate, NE: Norepinephrine, EPI: Epinephrine.

**Figure 5:**
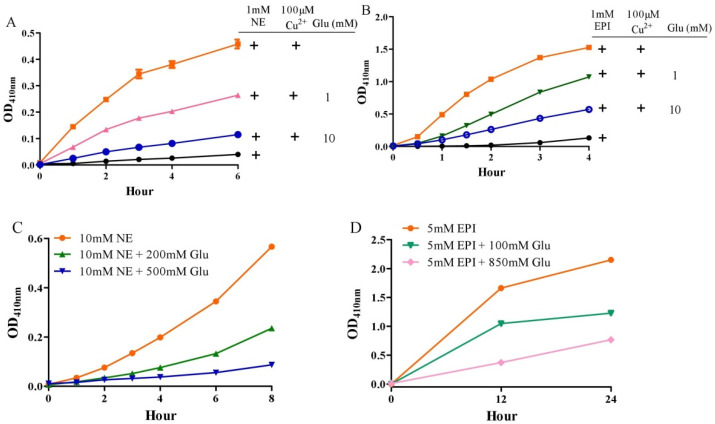
Influence of glutamate on norepinephrine or epinephrine oxidation. (**A**,**B**) Copper-promoted oxidation of norepinephrine and epinephrine, respectively, measured by OD_410 nm_. (**C**,**D**) Norepinephrine and epinephrine autoxidation, respectively, measured by OD_410 nm_. Chemicals were mixed in 0.15 M PBS (pH 7.4) and incubated at 37 °C for indicated time. Data are presented as mean ± range (*n* = 2). Note: Glu: Glutamate, NE: Norepinephrine, EPI: Epinephrine.

Since about 20% of copper is stored in the nucleus and copper is an essential component of chromatin [76], DNA is the primary target of copper/catecholamine-derived hydroxyl radicals [55]. As shown in Figure 4A, where lane 1 was the DNA prototype, 100 μM of dopamine perhaps had a weak damage effect (lane 2), and copper alone did not damage the DNA (lane 3). Dopamine-induced DNA damage was aggravated by copper in a dose-dependent manner (lane 6, 4). Specifically, while 10 μM of copper only initiated secondary DNA damage (lane 6), DNA degradation was almost completed by 25 μM of copper (lane 4). Under these conditions, −2.5 and 10 mM of glutamate, respectively, effectively inhibited the DNA damage (lane 7, 5). Similarly, when the dopamine was replaced with the same dose of norepinephrine or epinephrine (100 μM), copper was again found to promote DNA damage (Figure 4B, lane 2, and Figure 4C, lane 3). Again, glutamate dose-dependently inhibited the DNA damage (Figure 4B,C) and 10 mM of glutamate nearly completely inhibited the DNA damage (Figure 4B, lane 4, and Figure 4C, lane 6). The protective role of glutamate on dopamine/copper-triggered DNA damage could be attributed to the inhibitory effect of glutamate on copper-promoted dopamine oxidation, as demonstrated above. We thus inferred that the protective role of glutamate on norepinephrine or epinephrine/copper-triggered DNA damage is also associated with the inhibitory effect of glutamate on copper-promoted norepinephrine or epinephrine oxidation. This was validated by our next experiment. Copper at a level of 100 μM greatly promoted the oxidation of 1 mM of norepinephrine or epinephrine, as assessed by OD410nm (Figure 5A,B); however, as little as 10 mM of glutamate substantially suppressed the copper-mediated oxidation of the catecholamine (Figure 5A,B). Since the “autoxidation” of catecholamines should be iterated as the “trace transition metal-mediated oxidation” of catecholamines, and glutamate can restrain the “autoxidation” of dopamine by depriving the redox activity of copper, we thus hypothesized that glutamate ought to prevent norepinephrine or epinephrine from undergoing so-called autoxidation. Indeed, the oxidation of both norepinephrine (10 mM) (Figure 5C) and epinephrine (5 mM) (Figure 5D), as assessed by OD410nm, increased as a function of time; however, this was nearly completely inhibited by 500 mM and 850 mM of glutamate, respectively, according to the results shown in Figure 5C,D.

### 3.5. Glutamate Protects against the Toxicity of 3, 4-Dihydroxy Phenylacetaldehyde

**Figure 6:**
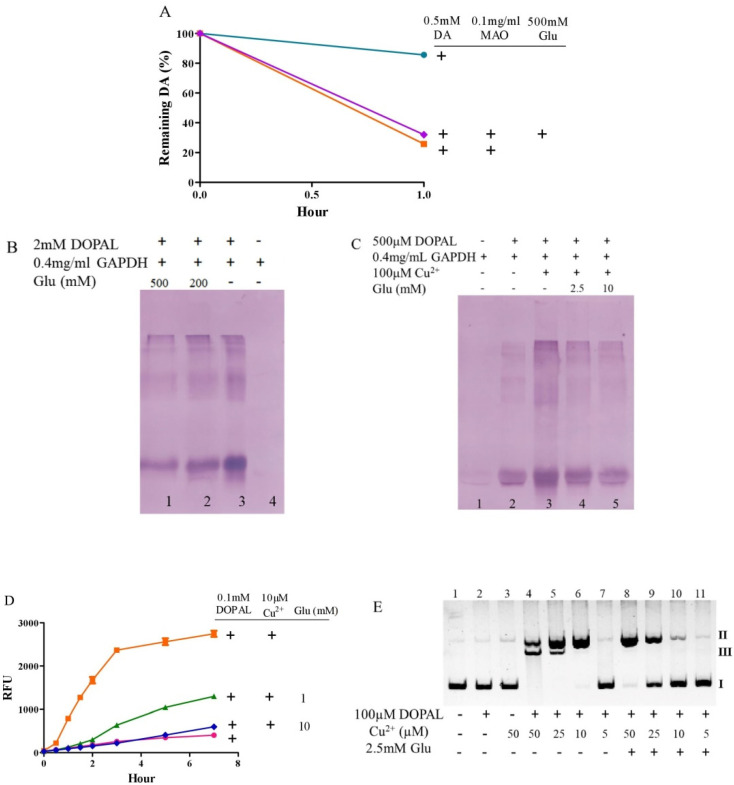
Glutamate attenuates side effects of DOPAL. (**A**) MAOmediated dopamine oxidation detected by HPLC. (**B**,**C**) DOPAL-caused quinoprotein in GAPDH, in the absence or presence of copper, respectively. (**D**) DOPAL-caused hydroxyl radical production detected by 3-CCA. (**E**) Copper/DOPAL induced DNA damage.Chemicals were mixed in 0.15 M PBS (pH 7.4) and incubated at 37 °C for indicated time or 1 h for B, 20 min for C and 30 min for E. Data are presented as mean ± range (*n* = 2). Note: DA: Dopamine, Glu: Glutamate.

MAO [77] catalyzes the oxidative deamination of dopamine to form DOPAL, which is then oxidized to DOPAL-quinone [78,79]. DOPAL, as the critical endogenous toxin causing dopaminergic neuron loss in Parkinson’s disease, is 1000-fold more toxic than dopamine in vivo [80]. DOPAL-induced protein modifications were enhanced by copper [81]. Oxidatively damaged DNA, caused by catecholamine-related neurotoxins, contributes to neuronal death [26]. Although glutamate could not inhibit the MAO-mediated oxidation of dopamine (Figure 6A), it was able to powerfully inhibit DOPAL-induced quinoproteins (Figure 6B), DOPAL/copper-enhanced quinoprotein formation (Figure 6C), and DOPAL/copper-produced hydroxyl radicals (Figure 6D). The copper-mediated oxidative toxicity of DOPAL was also observed in DNA. As shown in Figure 6E, DOPAL and copper alone did not damage the DNA (lanes 1–3). However, when 100 µM of DOPAL was added together with different concentrations of copper, copper-dependent DNA damage was observed: secondary damage with 10 μM of copper (lane 6) and tertiary damage with 25 and 50 μM of copper (lane 5 and 4, respectively). A protective effect of 2.5 mM of glutamate against copper-enhanced DOPAL damage to DNA was consistently observed at all the copper concentrations examined (lanes 10 vs. 6; lanes 9 vs. 5; lanes 8 vs. 4).

### 3.6. Considerations of Molar Ratio of Glutamate to Dopamine

Unlike neuromodulators such as dopamine and norepinephrine that show a marked regional distribution in the brain, glutamate is present at high concentrations in all cells via a high-affinity uptake system [35]. The human neuron-specific glutamate transporter (EAAT3) is densely expressed in dopaminergic neurons. The dense expression of EAAT3 in dopaminergic neurons not only detoxifies extracellular glutamate but also fulfills the high energy requirements of these cells, since glutamate participates in energy metabolism via glutamate dehydrogenase, which is also co-expressed in dopaminergic neurons [35]. It is known that the intracellular concentration of glutamate is approximately 10 mM [35] and that the dopamine concentration in dopaminergic neurons is at the range of 0.05–0.1 mM [37,38,39]; therefore, the molar ratio of glutamate to dopamine could reach 200 in dopaminergic neurons. It should be noted that the dopamine concentrations used in the various in vitro experimental systems were not identical in the present study. The major reason why we did not use the same physiologically relevant concentration of dopamine in each test system, including dopamine oxidation, dopamine-caused quinoprotein formation, and dopamine/copper-triggered DNA damage, was the different sensitivities of the in vitro experimental approaches. Nonetheless, we always observed the protective effect of glutamate on the pro-oxidant actions of dopamine under a molar ratio of 200. It should be stated that these experiments were not carried out under physiological conditions and may not be related to processes taking place in the brain.

## 4. Conclusions

Glutamate and catecholamines are essential neurotransmitters that are endowed with corresponding important physiological functions. Catecholamines are susceptible to undergoing non-enzymatic and enzymatic oxidation, and copper is involved in both types of oxidation. Catecholamine oxidation, along with the formation of ROS, highly reactive quinones, and toxic DOPAL (in the case of dopamine), has been implicated as a causative pathological mechanism in several neurodegenerative diseases, including Parkinson’s disease. For the first time, the present study demonstrates that the neurotransmitter glutamate, which is enriched and reaches a level 200-fold higher than that of dopamine (the major catecholamine) in terms of molar concentration in the brain, has a pronounced inhibitory effect on catecholamine oxidation in vitro when the molar ratio of glutamate/catecholamines is lower than 200, via the chelating and restraining redox activities of copper. 

## Data Availability

The data presented in this study are available in the article. Additional data are available on request from the corresponding author.
